# A systematic study of PPLN length dependence in intracavity SHG

**DOI:** 10.1038/s41598-021-01699-0

**Published:** 2021-11-15

**Authors:** Tyler Kashak, Liam Flannigan, Chang-qing Xu

**Affiliations:** Engineering Physics, McMaster University, 1280 Main Street West, Hamilton, ON L8S 4L8 Canada

**Keywords:** Applied optics, Lasers, LEDs and light sources, Optical materials and structures, Optical physics, Optical techniques

## Abstract

In this paper, a systematic study of the relationship between nonlinear crystal length and intracavity second-harmonic generation (SHG) using MgO-doped periodically-poled lithium niobate (MgO:PPLN) is presented. The experimental results demonstrate a relationship between the maximum SHG power generated and the full-width at half maximum (FWHM) of the crystal’s temperature tuning curve to the length of the nonlinear optical crystal. It was shown that maximum SHG power increases rapidly with the increase of MgO:PPLN length, reaching a saturation length (~ 2 mm), which is much shorter than that predicted by the single-pass SHG theory. This saturation length of the MgO:PPLN crystal is almost independent on 808 nm pump power for typical powers used in continuous wave intracavity SHG lasers. In addition to this saturation effect, a broadening effect was also observed, the FWHM of the temperature tuning curve was shown to have a larger FWHM than that predicted by the single-pass SHG theory for MgO:PPLN shorter than the saturation length. This work has the benefit of allowing engineers to optimize nonlinear crystal length when developing intracavity SHG based diode-pumped solid state (DPSS) lasers.

## Introduction

Diode-Pumped Solid-State (DPSS) lasers based on intracavity second harmonic generation (SHG) are highly desirable for a wide range of applications including laser displays^1–3^, biomedicine (microscopy and spectroscopy)^4,5^, optical communications^6^, and material processing^7,8^. In the past, such DPSS lasers were used for single-pass second harmonic generation configurations to produce continuous-wave (CW) visible lasers^9–17^. An intracavity structure is commonly used to increase the effective interaction length in the nonlinear crystals and the fundamental wave intensity. This approach has a number of benefits over the single-pass approach, such as compact form factor, low-cost, and a high conversion efficiency and high output power^18–29^.

Nonlinear crystals such as lithium triborate (LBO)^27–29^, potassium titanyl phosphate (KTP)^19–21^, periodically-poled potassium titanyl phosphate (PPKTP)^22,23^, and magnesium-oxide doped periodically-poled lithium niobate (MgO:PPLN)^24–26^ have been used in both the single-pass and intracavity SHG. Among these nonlinear crystals, MgO:PPLN has attracted a lot of attention due to its high nonlinear coefficient, large available crystal size and simple wafer-based fabrication process. It is estimated that the raw material cost is only ~ $2/mm for MgO:PPLN. A variety of crystal lengths have been reported for MgO:PPLN crystals in both the single-pass and intracavity SHG configurations. The intracavity structures included nonlinear crystal lengths between 1 and 10 mm, while the single-pass setups employed longer crystals from 10 to 50 mm. For example, MgO:PPLN crystals with lengths of 1, 2, 3, and 10 mm were used in an intracavity setup to achieve maximum SHG powers of 1.28, 2.8, 6.6, and 1.3 W respectively^1,24–26^. However, there has been limited discussion in terms of developing a clear relationship between maximum SHG power and MgO:PPLN crystal length.

It is clear from the reported results that crystal length is an important parameter in determining the output power of SHG lasers. There are also diminishing returns on increasing the crystal length, as the temperature tolerance of the SHG lasers decreases with the increase of crystal length and a longer crystal does not guarantee significantly higher SHG output power. As a result, a given laser cavity will have an optimum crystal length where the output SHG power of the laser effectively saturates. If the crystal length is increased past this “saturation length”, there will be no meaningful increase in power versus the increased cost of fabrication and size of the laser. The argument could be made that this is considered “common knowledge” by those who work with SHG lasers regularly. However, across all the reviewed literature, there was no evidence of an exhaustive study to determine the optimal crystal length for a given laser cavity. We expect that this saturation length will be a function of not only the type of nonlinear crystal used, but also the cavity configuration. There is also incentive to use shorter crystals, if possible, as the temperature tolerance for phase matching becomes stricter as the nonlinear crystal gets longer. This means that lasers with shorter crystals can be more robust and resistant to changes in environmental conditions over lasers with longer crystals. As a result, the main novelty of this paper is to present a systematic study of PPLN length versus maximum SHG output power for compact, watt-level green SHG lasers. Such lasers have strong demand in applications such as laser display and biomedical applications. This is an area our research group has worked on extensively^30,31^, and the simple cavity structure should serve as a good baseline example of how MgO:PPLN length and fundamental pump power modify the expected maximum SHG output power.

MgO:PPLN is an attractive option for the low-cost, compact watt-level lasers that our research group has worked on for laser displays and biomedical applications^1,3^. This is a result of MgO:PPLN fabrication to produce long crystals with sufficiently large 0.5–1 mm aperture sizes relative to the beam diameters inside the laser cavity, which are typically < 100 microns. The wide body of research available as seen from the previously cited works for MgO:PPLN reinforces the importance of developing empirical relations that can guide the design and optimization of MgO:PPLN-based lasers. Additionally, such relations may be able to generalize to multiple nonlinear crystals, increasing their overall utility and simplifying the design process for SHG lasers.

In this paper, a compact, continuous wave (CW), intracavity SHG laser based on MgO:PPLN is systematically studied. Several bulk MgO:PPLN crystals of varying lengths were used to examine the impact on intracavity SHG laser performance. Parameters such as the maximum SHG power (i.e., SHG power at the optimal quasi-phase matched (QPM) temperature) and full width at half maximum (FWHM) of the temperature tuning curve were examined. In addition to varying the nonlinear crystal length, the 808 nm pump power was also varied to illustrate the dependence on pump power. It was shown that the length of the MgO:PPLN crystal where maximum power saturation occurs is consistent across pump powers, and that crystal lengths greater than 2 mm offer little additional SHG output power. In this case, we would state that the saturation length of MgO:PPLN crystal for this cavity configuration is 2 mm, and crystal lengths greater than the saturation length offer minimal benefits. The maximum SHG power versus PPLN crystal length is found to follow a logarithmic function, and an empirical relation for the coefficients and intercept of the logarithmic function are derived. This relation can potentially be used with similar cavity structures to derive an optimal MgO:PPLN length for a given laser cavity, cutting down on fabrication costs by allowing smaller MgO:PPLN crystals to be used for equivalent SHG output powers. The derived empirical relation and experimental results provide an optimized guideline for the use of MgO:PPLN in DPSS SHG lasers such that large temperature tolerance and high maximum SHG powered can be coupled with the reduction of cost and overall laser cavity complexity.

## Materials and methods

**Figure 1:**
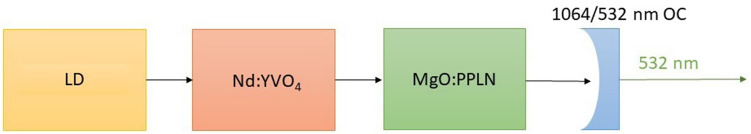
Rough schematic of the LD end-pumped DPSS 532 nm green laser.

The experimental setup of the end pumped intracavity frequency doubled Nd:YVO_4_/MgO:PPLN green laser at 532 nm is shown in Fig. 1. This is a plano-concave cavity design to enhance the simplicity of the straight cavity and reduce the number of optical elements by using the back surface of the Nd:YVO_4_ crystal as one of the mirrors. The pump source was a 10 W F-mount 808 nm laser diode (LD). The fundamental laser crystal was a 3 × 3 × 5 mm^3^ a-cut Nd:YVO_4_ crystal with an Nd doping concentration of 1.1 atm%. The input facet of the laser crystal was anti-reflection (AR) coated at 808 nm and high reflection (HR) coated at 1064 nm to act as one of the end mirrors forming the laser cavity. The output facet of the Nd:YVO_4_ was AR coated at 1064 nm to minimize loss at the fundamental wavelength. The 5 mol% MgO:PPLN had an aperture of 0.5 × 2 mm^2^ with a uniform domain inversion period of 6.96 µm. Both facets of the MgO:PPLN were AR coated at 1064 nm and 532 nm. To optimize and further examine performance, the LD, Nd:YVO_4_, and MgO:PPLN were attached to the cool side of thermo-electric cooling (TEC) devices. A plano-concave output coupling (OC) mirror with a radius of curvature of 100 mm and diameter of 6 mm was used to complete the intracavity structure. The OC mirror was HR coated at 1064 nm and high transmittance (HT) coated at 532 nm. The overall length of this straight cavity remained approximately 40 mm for all experiments.

## Results

The experimental results were obtained by selecting several MgO:PPLN samples with lengths ranging from 1 to 4 mm. The temperature of the MgO:PPLN was varied in $1^\circ$C increments for all experiments outside of the 3 and 4 mm MgO:PPLN crystals to achieve optimal phase matching and generate temperature tuning curves. For the longer 3- and 4-mm crystals, the temperature tuning curve was narrow enough that steps of 0.25 °C were used to maintain a high resolution where the maximum SHG power could be readily observed. The maximum SHG power was recorded at the QPM temperature that corresponded to the highest 532 nm green output power for each crystal. The temperature tuning curve was then used to calculate the FWHM (also referred to as temperature tolerance or temperature bandwidth) for the corresponding sample. This process was repeated for multiple values of 808 nm pump power (2, 3, 4 and 5 W) using the same MgO:PPLN samples over multiple trials to obtain this relationship.

### Maximum SHG power vs. PPLN length

**Figure 2:**
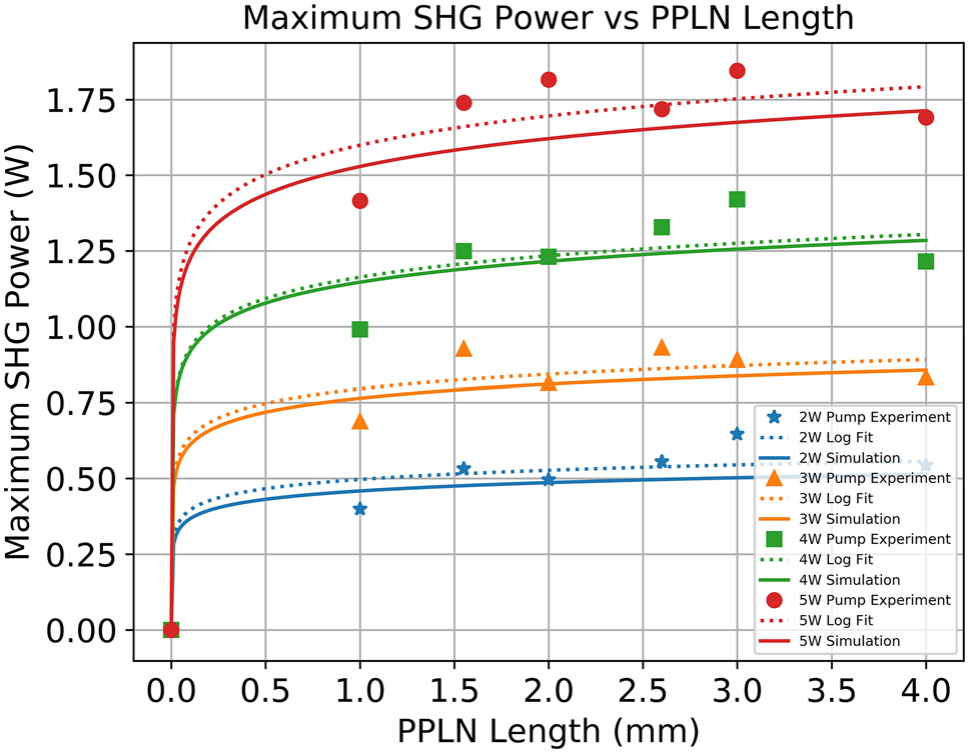
Measured maximum SHG power vs. PPLN length for several values of 808 nm pump power.

**Figure 3:**
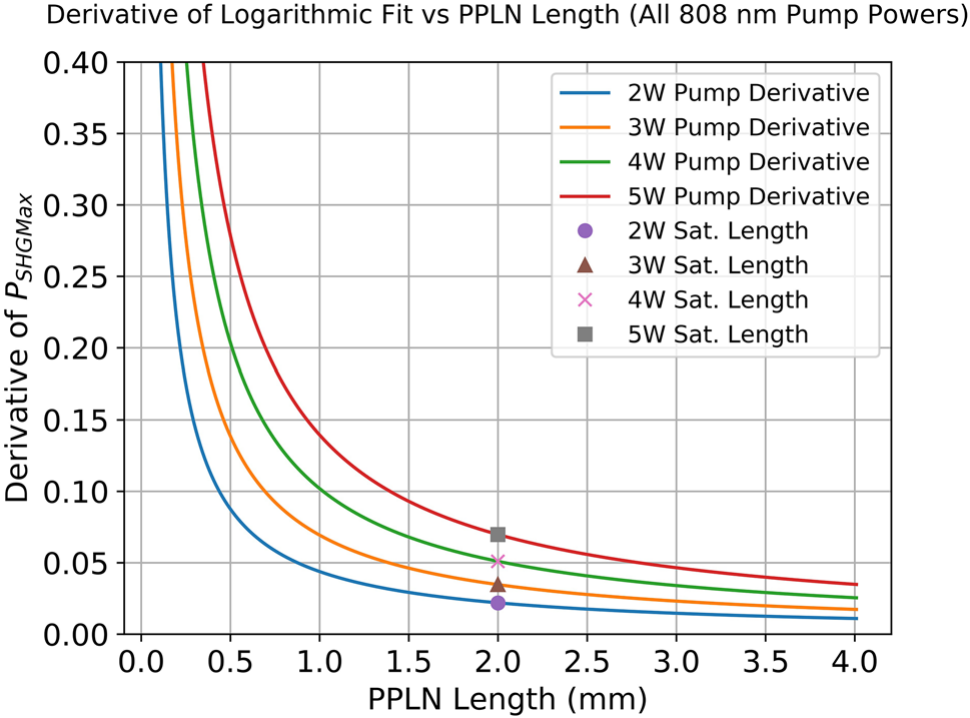
The derivative of the logarithmic fit to the maximum SHG power vs. PPLN length for several values of 808 nm pump power. The markers denote the saturation length to be 2 mm for all pump powers.

Figure 2 shows the measured maximum SHG power as a function of MgO:PPLN length. In Fig. 2, the x-axis represents device length, and the y-axis represents the maximum SHG power. The 2 W, 3 W, 4 W, and 5 W 808 nm pump powers are represented by stars, triangles, squares, and circles, respectively. The dotted lines are a logarithmic fit to the data to illustrate the observed trend. The solid lines represent the theoretical prediction from a single longitudinal mode intracavity simulation developed by our research group (which will be discussed in more detail later in this section). The dotted lines are logarithmic fits to the experimental data meant to highlight the overall trend of maximum SHG power vs MgO:PPLN length. These logarithmic fits are used to derive an empirical relation that links the 808 nm pump power to the maximum SHG power as a function of MgO:PPLN length and are described in more detail in “Empirical relation for SHG power vs. length” section. There are several important features to point out in the results. First, the measured maximum SHG power increases with the MgO:PPLN length and reaches a saturation point where increasing the length of the nonlinear crystal leads to diminishing returns on increasing the maximum SHG power. We have defined the length where this saturation begins (the “saturation length” of the crystal) to be the point where the derivative of the Maximum SHG Power vs PPLN Length function is equal to 5% of its value for 0.1 mm long PPLN. Using this definition, the saturation length was found to be 2 mm for all pump powers. This can be observed in Fig. 3. Figure 3 shows the derivative of the logarithmic fit for Maximum SHG Power vs PPLN length at all pump powers. In Fig. 3, the x-axis represents device length, and the y-axis represents the first derivative of the maximum SHG power. The saturation length for each pump power is marked with a single data point, with the circle, triangle, cross, and square representing the 2 W, 3 W, 4 W, and 5 W saturation length, respectively. The logarithmic fits used to produce these derivatives are detailed further in “Empirical relation for SHG power vs. length” section. Using the definition of saturation length provided previously, we can make the second observation that the saturation length does not vary with pump power under this definition. This makes sense, as the derivative of the logarithmic fitting functions are $1/x$ curves that only vary by a multiplicative constant. As a result, the saturation length is constant for this range of pump powers. Another reason for selecting this definition for saturation length is that it provides an easily testable metric against other nonlinear crystals and cavity configurations for future work.

To verify that the saturation length and logarithmic trend extended to other intracavity SHG lasers, we simulated a 561 nm laser based off the 1122 nm laser line in Nd:YAG. It was found that this laser had significantly lower output power compared to the 532 nm laser thanks to the lower gain of this laser line, which is discussed in more detail later in this section. However, the saturation length remained the same at 2 mm, showing that the gain of the laser medium is not a contributing factor to the saturation length. A future investigation will look at various parameters of the laser cavity to determine the underlying physics that govern the saturation length of a nonlinear crystal. To investigate the underlying physics behind the observed features, the SHG power dependence on MgO:PPLN length was simulated using an intracavity SHG theoretical model, with the results plotted in Fig. 2. This figure presents the simulated results for maximum SHG power (y-axis) versus MgO:PPLN length (x-axis) for an 808 nm pump power of 2, 3, 4 and 5 W (solid lines). The simulation was developed in-house by our research group and has been reported in a previous publication^32^. While the model is described in full detail in the cited publication, we will briefly summarize the model here. The model uses the coupled rate equations for a four-level gain medium in a laser cavity to determine the circulating fundamental 1064 nm power in the cavity. This is affected by both the linear loss due to imperfect optical coatings, scattering, absorption, and similar phenomena, as well as an additional nonlinear loss induced by the nonlinear crystal when the fundamental 1064 nm light is converted into 532 nm light. The steady state fundamental power from the cavity equation was used to estimate the generated forward and backward SHG power using the coupled nonlinear wave equations as derived by Boyd^33^. This generated SHG power was used to update the nonlinear loss term, and the rate equations are used to generate a new steady state power. This process was repeated until the steady state fundamental power, output SHG power, and nonlinear loss are no longer changing between each iteration. The temperature dependent Sellmeier equations were used to generate a temperature tuning curve using the described model. For each theoretical curve in Fig. 2, the MgO:PPLN length and pump power was set to the appropriate value, and the model runs until convergence. The linear loss was calculated for each individual MgO:PPLN based on the designed AR coating of the crystal and the reflectivity of the output coupler. The leaking fundamental power can be estimated in the simulation by taking the calculated steady state intracavity power and multiplying by the transmission of the OC mirror. For a list of the typical loss values and other simulation parameters used in the intracavity model, please refer to Ref.^32^. The circulating fundamental power inside the cavity was measured experimentally by performing the reverse operating, where the measured leaking power was used alongside the transmission of the OC to estimate the intracavity power. The theoretical results were found to be in good agreement with the experimental results. For a more in-depth discussion on the theoretical model, refer to Ref.^32^.

It can be observed that our theoretical model in Fig. 2 also follows a logarithmic trend matching the experimental data, although the maximum SHG power is consistently lower (~ 2–5%, depending on pump power) than the experimental trend line. Ignoring experimental error, this can be attributed to the plane-wave assumption of the model as well as the single mode assumption. The intracavity green laser presented here lacks any wavelength selection elements (such as an etalon). As a result, it is almost certainly a multi-longitudinal mode laser. Additionally, the plane wave assumption of the model means that the Gaussian nature of the light in the cavity is overlooked. Therefore, effects such as beam overlap and focusing in the PPLN crystal are not considered in the modeling. Despite these drawbacks, the overall logarithmic trend, and the relatively good agreement of the theoretical prediction with the experimental trend shows that these effects are not major effects that must be considered with our laser. While the model would most certainly be improved by adding modeling for multiple modes and Gaussian propagation, this work, and the work in Ref.^32^ show that reasonably close results can be obtained for the maximum SHG power using a simplified single mode model that requires significantly less computational power. Additionally, the lack of wavelength selection device in our laser can be attributed to our target application, which is low-cost, compact, watt-level lasers that can be manufactured easily. Etalons are difficult to align and introduce additional linear losses to the laser that decrease the potential output power, and our previous work has shown that the stability without an etalon can be kept below 5%, which is acceptable for laser displays and other applications. A discussion on the stability of the laser as well as the effects of introducing a wavelength selection component are beyond the scope of this paper.

**Figure 4:**
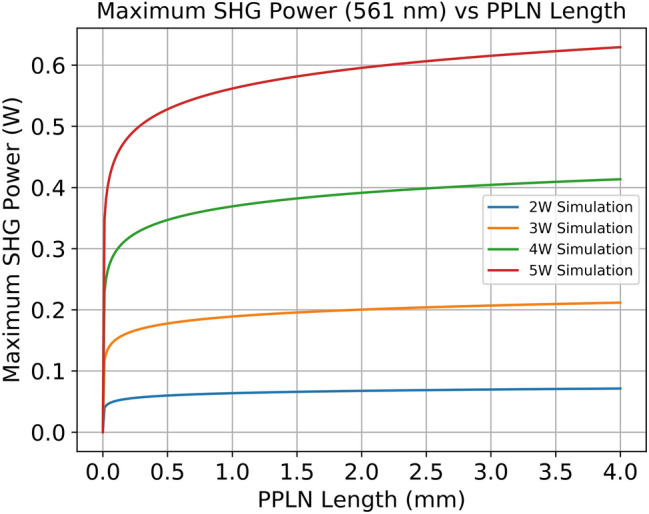
Simulated maximum SHG Power at 561 nm vs. PPLN length for several values of 808 nm pump power.

The simulation was also extended to a 561 nm SHG laser utilizing the 1123 nm transition found in Nd:YAG. This wavelength has several uses in the biomedical sciences, having favorable absorption properties in materials such as hemoglobin and less heat dispersion in the retina over comparable lasers^34^. This laser was selected due to the significantly lower gain of the 1123 nm line, as the stimulated emission cross section of the 1123 nm line is only ~ 1/16th that of the 1064 nm line at $3\times {10}^{-19}\mathrm{ c}{\mathrm{m}}^{2}$^35^. We wished to see if similar logarithmic behavior and saturation lengths could be predicted. There is no experimental data to accompany this simulation however, due to a lack of PPLN samples with varying lengths and the appropriate poling period for 561 nm generation. Instead of fitting a logarithm to the experimental data for 561 nm SHG, instead the simulation data was used to fit a logarithm and calculate the derivative of the fit for the saturation length. The maximum SHG power vs. PPLN crystal length is presented in Fig. 4, where the x-axis is the PPLN length in mm and the y-axis is the output 561 nm SHG power in Watts. Each line represents a different value of 808 nm pump power, covering 2 W, 3 W, 4 W, and 5 W.

We can clearly see the same logarithmic trend in the data as observed in Fig. 2. The main difference is the maximum power achieved, which is significantly lower than the 532 nm SHG. If we consider the 4 mm crystal at 5 W pump, the 532 nm power was ~ 1.65 W, while the 561 nm power for the same conditions is ~ 650 mW. This represents a reduction of almost 60%, which can be attributed to the much lower gain of the 1123 nm laser line. As a result, the circulating power within the cavity is much more susceptible to losses induced by both imperfect optical coatings and the nonlinear losses induced by the SHG process, significantly reducing the possible output power. Nonetheless, the logarithmic trend is maintained in both the 532 nm and 561 nm lasers.

The logarithmic trend can be explained by the physics demonstrated in the model from Ref.^30^. As the PPLN length is increased, the efficiency of the SHG process increases, and so we see an increase in maximum SHG power. However, these gains saturate quickly once the saturation length is reached. This occurs as the increased SHG conversion efficiency from longer crystals is balanced by an equivalent drop in circulating fundamental 1064 nm power due to the increased nonlinear loss in the cavity. Basically, past a certain point, any gain in SHG efficiency is counterbalance by a drop in circulating 1064 nm power that negates most of the benefit of the longer crystal. Therefore, there exists a self-stabilization effect where any increase in the conversion efficiency of the nonlinear crystal has no effect on SHG maximum power as there is always an equal and opposite drop in the fundamental wavelength power. This general trend holds at higher pump powers, showing that while the overall SHG output power increases with 808 nm pump power due to the increased circulating 1064 nm power, the self-stabilization occurs around the same length of PPLN as it does at lower crystal lengths. As a result, for a given range of 808 nm pump powers, there exists an optimal saturation length where there are negligible increases in maximum SHG power when using crystals longer than the saturation length. This matches the general theory developed by Smith which shows that for a given cavity and PPLN length, which draws similar conclusions about how fundamental wave depletion in intracavity structures limits the generation of maximum SHG power^33^. This allows the optical engineer to design a laser cavity with no “wasted” space, and in “Empirical relation for SHG power vs. length” section we derive empirical relations to quantify this optimization process in an easy-to-understand format. These findings can act as guidelines when developing a compact and high-performance DPSS SHG laser structure.

### FWHM vs. device length

**Figure 5:**
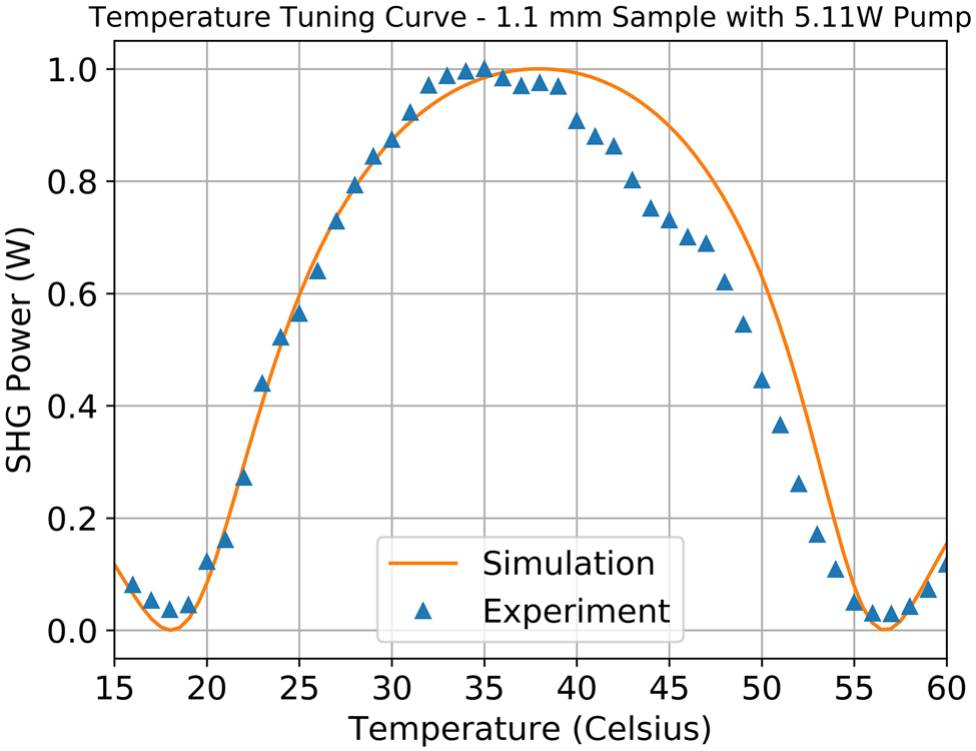
Normalized temperature tuning curve at 5 W 808 nm pump power vs. simulated temperature tuning curve using the intracavity theory.

Figure 5 illustrates the normalized SHG power (y-axis) as a function of PPLN temperature (x-axis) for both the collected experimental results as well as the previously explained theoretical intracavity SHG model. The triangles represent the experimental data at a pump power of 5 W, while the solid line represents the simulated results for an intracavity SHG temperature tuning curve at a pump power of 5 W. The experimental and theoretical results are for a MgO:PPLN sample with a length of 1 mm. A temperature sweep was performed (sweeping from low to high temperatures and high to low temperatures and taking the average to mitigate hysteresis effects) using steps of 1 °C to generate a curve revealing the temperature bandwidth of the sample. For the longer 3- and 4-mm crystals, the temperature tuning curve becomes narrow enough that steps of 1 $^\circ$C no longer provided sufficient resolution to be confident of the peak of the temperature tuning curve. As a result, the 3- and 4-mm crystals had temperature tuning curves generated using a step size of 25 °C to maintain sufficient resolution. This process was repeated for multiple values of 808 nm pump power (2, 3, 4, and 5 W) using MgO:PPLN samples with different lengths over multiple trials to ensure repeatability of the measured data. Due to the similarity between the various temperature tuning curves, we have only included the 5 W data in the figure as an illustrative example.

As expected, the maximum SHG power is achieved at the optimal QPM temperature. Also, from Fig. 5, it can be observed that the intracavity model provides a reasonably good fit to the experimental data, with the theoretical and experimental FWHM only differing by ~  2°. The simulated results slightly overestimate the width of the temperature tuning curve when directly compared to the experimental data for the intracavity SHG. This feature is likely a result of a known issue with our TEC controller, which is hard to maintain a uniform temperature at temperatures above 30°. This lack of symmetry to the temperature tuning curve could also be a result of manufacturing error with the MgO:PPLN sample.

**Figure 6:**
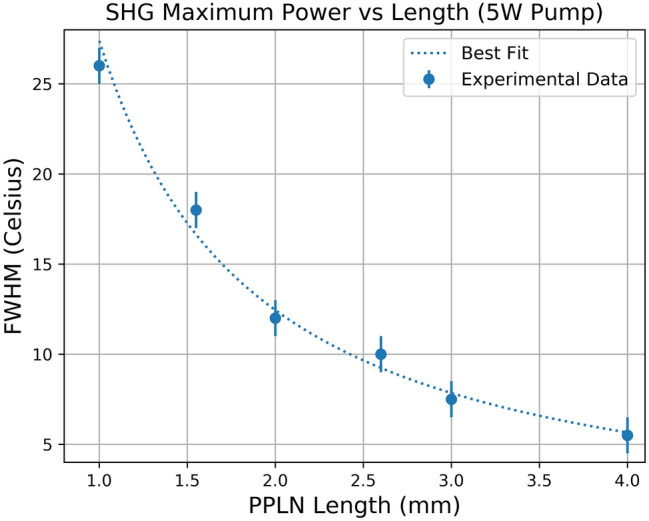
Measured FWHM of temperature curve vs. PPLN length at 5 W 808 nm pump power. The dotted line represents the negative power fit to the data, showing that as the PPLN length increases the FWHM decreases more slowly than at shorter lengths.

It is worth noting that the FWHM from the intracavity SHG shown in Fig. 5 is 30 °C, which is much broader than that predicted from the single-pass SHG theory (18 °C). The broadening effect of the FWHM in the intracavity SHG can be understood as follows: the nonlinear loss at non-ideal operating temperatures (away from the satisfied QPM condition) is lower due to reduced phase-matching efficiency. This reduction in cavity loss results in an increase of circulating fundamental power within the cavity as a result, the output SHG power is larger than expected at non-ideal temperatures as the decrease in nonlinear loss from non-ideal phase matching is partially offset by a higher circulating fundamental power. This broadening of the temperature bandwidth represents an increase in overall temperature tolerance and thus power stability for intracavity SHG as compared to single-pass SHG. It is also shown below in Fig. 6 that the FWHM of the temperature tuning curve decreases rapidly with increasing MgO:PPLN length. Thus, the laser designer should try to balance the improved efficiency of longer MgO:PPLN crystals with the wider temperature tolerance (and resulting insensitivity to environmental conditions) found in shorter MgO:PPLN crystals.

Figure 6 shows the measured FWHM of the intracavity SHG (y-axis) as a function of MgO:PPLN length (x-axis) at a 5 W pump. The uncertainty associated with the FWHM was ± 0.5 °C. It is evident that the device length has an important impact on the FWHM of the temperature tuning curve.

The experimental results of Fig. 6. show a clear decrease of the FWHM of the temperature tuning curve with increasing nonlinear crystal length. This is the result of two phenomena: phase mismatching accumulation and beam nonuniformity. The phase mismatching accumulation refers to how it is difficult to ensure quasi-phase matching for a longer nonlinear crystal than it is for a shorter nonlinear crystal. The difficulty of maintaining a uniform temperature over large crystals versus smaller crystals also contributes to the reduction of the FWHM. The beam nonuniformity refers to the fact that the beam diameter is not uniform within the nonlinear crystal. The longer the nonlinear crystal, the higher the impact of the beam nonuniformity to second harmonic generation efficiency. As shown in Fig. 6, the FWHM decreases more quickly before the saturation length (< 2 mm) and proceeds to decrease more slowly after the saturation length (> 2 mm). It is worth noting that a similar trend has been observed in the FWHM for the intracavity SHG simulations. The dotted line represents a negative power fit to the data meant to illustrate the rate of change of the FWHM with PPLN length more clearly.

When developing a laser which will be subject to temperature fluctuations, the power stability is dependent upon the length of the nonlinear crystal governing the SHG process. This is vitally important when selecting an optimized device length as there is a clear trade-off between maximum SHG power (longer device) and a larger FWHM for maximum temperature stability (shorter device).

### Empirical relation for SHG power vs. length

The experimental data for the maximum SHG power as a function of PPLN length in Fig. 2 was fit using a logarithmic function. This fit was selected as both the experimental data and the theoretical prediction from the intracavity SHG model appeared to be logarithmic in nature. A general logarithmic relation was derived:

1 $${P}_{SHGmax}=a\cdot \mathrm{ln}\left({l}_{c}\right)+b.$$

With the results of the logarithmic relation for each value of 808 nm pump power listed in Table 1 below for 532 nm emission below. The 561 nm coefficients are not listed, but were similar to the 532 nm coefficients, with the only major difference being the reduction in the coefficient due to the lower overall SHG power in the 561 nm case.where ${P}_{SHGmax}$ is the expected maximum SHG power (in units of Watts) and ${l}_{c}$ is the PPLN length (in units of mm). There are two components to each logarithmic equation: the coefficient for the natural logarithm, and the intercept when the length is zero. This is somewhat misleading as the natural logarithm of zero is undefined, and so 1E-5 was used as the “zero” length for the PPLN crystal instead.

**Table 1:** The value of the coefficient and intercept of the logarithmic relation for 532 nm that was obtained for each value of 808 nm pump power in Watts.

808 nm pump power (W)	Value of a coefficient	Value of b intercept
2	0.0438	0.4966
3	0.0693	0.7952
4	0.1019	1.1623
5	0.1394	1.5992

**Figure 7:**
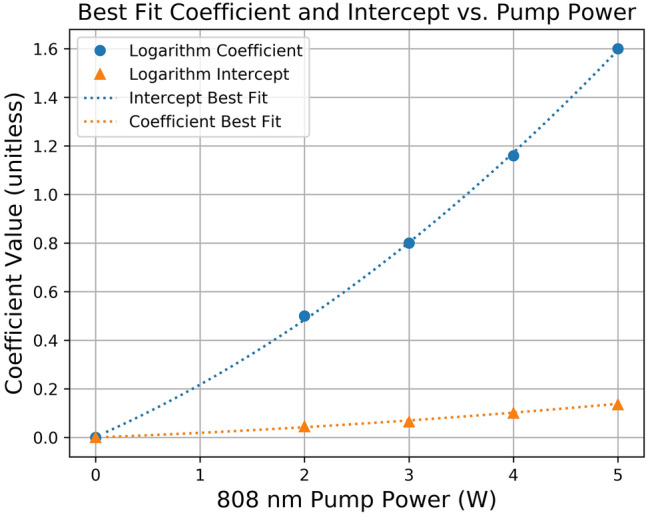
The coefficient and intercept values for the 532 nm logarithmic relation derived using experimental data for maximum SHG power vs. PPLN length.

We wish to determine if there is a relation that can be used to predict the coefficient and intercept of the logarithmic equation as a function of 808 nm pump power. The coefficient and intercept were plotted and fit with a second order polynomial, which resulted in an ${R}^{2}$ value of 1 for both fits. The results can be seen in Fig. 7, where the x axis represents the 808 nm pump power (in Watts), while the y axis represents the numerical value of the coefficient/intercept. The circles represent the value of the logarithm coefficient, and the triangles represent the value of the intercept. The best fit equations are as follows:

2 $$\mathrm{Intercept}:\mathrm{ b}=0.0259{P}_{pump}^{2}+0.1888{P}_{pump}+0.0031,$$

3 $$\mathrm{Coefficient}: a=0.0022{P}_{pump}^{2}+0.0167{P}_{pump}+0.0003.$$

We can see that while there are some minor differences between the two relations, they both show a similar quadratic trend with the values of the coefficient and intercept. Ideally, we would be able to fit more data at higher pump powers to see if the overall trend holds, but we were limited to pump powers up to 5 W due to our limited selection of 808 nm diodes. This empirical relation potentially allows an optical engineer to select the operating pump power and derive a logarithmic relation that describes the SHG output power as a function of PPLN length. This could then be used to determine the optimal PPLN length for a given pump power, or the required pump power for a given desired SHG output power. Future work could expand on this and see if a similar relation can be found for other laser cavity configurations and higher pump powers.

## Conclusion

A systematic study on the relationships between maximum SHG power and the FWHM of the temperature bandwidth vs. nonlinear crystal length for MgO:PPLN has been presented. There are three main conclusions presented in this paper. First, it has been found that a saturation of the maximum SHG power occurs for an MgO:PPLN length of ~ 2 mm for 808 nm pump powers between 2 and 5 W. This serves as the optimum MgO:PPLN length for compact, watt-level intracavity green SHG lasers. Second, the operating temperature tolerance at the saturation length is ~ 10 $^\circ$C, which can be easily controlled using the conventional TEC controllers versus the stricter temperature requirements found in longer crystals. Finally, a novel set of empirical relations has been derived showing a logarithmic relationship between the maximum SHG power and the MgO:PPLN length. Additionally, a second relation is derived such that the 808 nm pump power can be used to derive the coefficient and intercept for the logarithmic relation for arbitrary pump powers. These relations can be used to determine the optimum MgO:PPLN length for potentially any pump power, or the required pump power and MgO:PPLN length for a desired SHG output power. Future work involves testing and understanding the physics of this relation for multiple nonlinear crystals to see if a more general empirical relation or figure of merit can be derived.
